# Immune profiling-informed immunomodulation associated with gestational extension in early-onset preeclampsia with monochorionic twins: a case report

**DOI:** 10.3389/fimmu.2026.1742646

**Published:** 2026-03-10

**Authors:** Dengqin Ma, Lina Zhang, Huyan Huo, Xiangyuan Liu, Sheng-Guang Li, Qun Li

**Affiliations:** 1 Department of Obstetrics and Gynecology, The First People’s Hospital of Tianshui, Tianshui, Gansu, China; 2 Department of Rheumatology and Immunology, Peking University International Hospital, Beijing, China

**Keywords:** certolizumab pegol, early-onset preeclampsia, hydroxychloroquine, immunomodulation, maternal–fetal tolerance, TNF-α, twin pregnancy

## Abstract

**Background:**

Early-onset preeclampsia (EOPE) is associated with placental malperfusion and systemic endothelial dysfunction. Maternal immune dysregulation has been implicated in EOPE, but immune findings obtained during severe disease are descriptive and do not define a validated EOPE subtype.

**Case presentation:**

A 22-year-old woman (G2P0) with a monochorionic diamniotic twin pregnancy developed gestational hypertension at 25^+5^ weeks and met criteria for EOPE at 30–31 weeks. Because of refractory progression under standard management, immune testing obtained at 31^+1^ weeks showed elevated TNF-α, low complement C4, weak ANA positivity, and T/B-cell imbalance (descriptive findings in this single case). After multidisciplinary discussion and informed consent for off-label therapy in pregnancy, an immunomodulatory regimen was started at 31^+1^ weeks (prednisone 10 mg/day, hydroxychloroquine 200 mg bid, and a single 200 mg SC dose of certolizumab pegol). Over the subsequent week, proteinuria decreased by >50% (10.78 to 4.99 g/24 h), blood pressure improved to 100–120/60–80 mmHg, and edema regressed. Gestation continued until 35^+0^ weeks, when cesarean delivery was performed for fetal compromise (Type II sFGR with umbilical-artery AEDF). Placental pathology showed multifocal infarctions and basal-plate fibrinoid deposition.

**Discussion:**

EOPE likely involves heterogeneous pathways in which inflammatory mediators, complement dysregulation, and cytokine excess have been reported. This case is hypothesis-generating and highlights the need for validated immune/angiogenic stratification and prospective evaluation of any adjunctive immunomodulatory approach in severe EOPE.

**Conclusion:**

In this single MCDA twin pregnancy complicated by severe EOPE and concurrent immune-activation markers, initiation of a pregnancy-compatible immunomodulatory regimen was temporally associated with maternal clinical stabilization and a 3–4-week interval to delivery for fetal indications. Controlled studies with validated phenotyping, serial biomarkers (including sFlt-1/PlGF), standardized endpoints, and detailed safety follow-up are needed before attributing benefit or recommending this approach.

## Introduction

Early-onset preeclampsia (EOPE), defined as onset before 34 weeks, is a leading cause of maternal and perinatal morbidity (1). It is thought to involve inadequate spiral artery remodeling, leading to placental malperfusion and the release of inflammatory and antiangiogenic mediators that contribute to systemic endothelial dysfunction (2, 3). Increasing evidence implicates maternal immune dysregulation—particularly exaggerated activation of innate immunity and imbalance in adaptive responses—in EOPE pathogenesis. Elevated pro-inflammatory cytokines such as TNF-α and reduced regulatory T cells are common findings, alongside complement activation and autoantibody presence in select patients (4).

Twin pregnancies, especially monochorionic diamniotic (MCDA), exacerbate immune and vascular stress due to larger placental mass and antigen exposure, leading to a 2–3× higher preeclampsia risk (5). Despite prophylaxis (e.g., aspirin), therapeutic options remain limited once EOPE is established (6). Recent studies suggest immunomodulatory agents—including hydroxychloroquine (HCQ) and TNF-α inhibitors—may be associated with clinical stabilization in selected autoimmune-related or inflammatory contexts (7). Certolizumab pegol, a PEGylated anti–TNF-α Fab′ fragment with minimal placental transfer, has shown promise in refractory obstetric autoimmune syndromes (8).

We report a severe EOPE case in an MCDA twin pregnancy in which immune testing (obtained during the severe disease stage) informed an immunomodulatory regimen after multidisciplinary discussion. This case provides hypothesis-generating observations and underscores the need for prospective, stratified studies before drawing causal conclusions about immunotherapy in EOPE.

## Case presentation

### Patient and obstetric background

A 22-year-old woman (G2P0) conceived monochorionic diamniotic (MCDA) twins spontaneously. She was generally healthy, with no prior hypertension, diabetes, or autoimmune diagnoses; notable history included a first-trimester pregnancy loss (February 2024) managed with curettage (no genetic testing). Menstrual cycles were regular (7/26 days). Pre-pregnancy/early-pregnancy features included microcytic anemia and hypertriglyceridemia. She did not smoke or drink. Last menstrual period was May 16, 2024; estimated due date February 23, 2025.

### Early pregnancy (6–22 weeks)

At 6^+4^ weeks, ultrasound confirmed a viable MCDA twin gestation; uterine artery Doppler showed high resistance (left S/D 6.5; right S/D 7.4) and a small intrauterine fluid collection. Prophylactic low molecular weight heparin (LMWH) and Chinese herbal therapy were used in early first trimester. At 9^+2^ weeks, embryos measured 2.5 cm and 2.6 cm with cardiac activity; uterine artery resistance remained asymmetric (left S/D 6.99; right S/D 3.5). At 12^+6^ weeks, NT was normal for both fetuses (1.6 mm), but the placenta was low-lying with a small subchorionic collection; anemia (Hb 107 g/L) and hypertriglyceridemia were noted, and iron supplementation was started. At 16^+2^ weeks, ultrasound suggested marginal/velamentous-like cord insertion for Twin B (During the prenatal period, the fetuses were designated by the ultrasound team according to fetal position in the uterus—the upper fetus was labeled “Twin A” and the lower fetus “Twin B”). At 17^+6^ weeks, non-invasive prenatal screening was low risk; at 22^+2^ weeks, both fetuses had single-loop nuchal cords, and a trivial pericardial effusion was seen on fetal echo.

### Onset of hypertension (25–28 weeks)

At 25^+5^ weeks, the first elevated blood pressure (BP) was recorded (147/96 mmHg; repeat 133/90 mmHg); she was asymptomatic and started on labetalol 100 mg bid, aspirin 75 mg qd, vitamin D, and iron. By 28^+2^ weeks, home BPs remained 140–150/90–100 mmHg, prompting an increase to labetalol 100 mg tid.

### First hospitalization (28^+6^ to 29^+4^ weeks; 2024-12-04 → 12-09)

She presented with worsening hypertension (147/96 mmHg), BMI 33.3 kg/m², and bilateral pitting edema (++). Key labs: Hb 99 g/L, Hct 32.2%, ferritin 8.6 ng/mL, total protein 61.0 g/L, albumin 34.1 g/L, triglycerides 5.74 mmol/L, total cholesterol 6.62 mmol/L, BNP 382.6 pg/mL, urine protein ++ with 24-h protein 0.16 g, and hypercoagulability markers TAT 21.7 ng/mL and D-dimer 3.03 mg/L. Ultrasound (29^+0^ weeks) showed normally grown twins: Twin A (cephalic) ~1375 g; Twin B (breech) ~1184 g; umbilical and middle cerebral artery Dopplers and uterine artery resistance were normal; cervix 39 mm. Ambulatory BP averaged 135/86 mmHg (peaks to 161/106 mmHg). Echocardiography showed a small pericardial effusion with preserved function. She was diagnosed with early-onset preeclampsia (EOPE) and treated with magnesium sulfate (5 g IV load; 1.5 g/h infusion), dexamethasone for fetal lungs (6 mg IM q12h ×4), intensified antihypertensives (labetalol 100 mg q8h), and aspirin 100 mg qd + fondaparinux 2.5 mg qd for thromboprophylaxis; iron therapy continued. BP stabilized and she was discharged on 12-09 with labetalol 100 mg bid, aspirin 100 mg qd, fondaparinux 2.5 mg qd, and iron.

### Second hospitalization (29^+6^ to 32^+0^ weeks; from 2024-12-11)

Two days post-discharge, she re-presented with BP 140/94 mmHg and progression of edema (legs and abdominal wall). Examination showed fundal height 37 cm; fetal heart rates 138/148 bpm. Laboratory worsening was evident: Hb 90 g/L, total protein 57.0 g/L, albumin 37.9 g/L, 24-h urine protein 6.70 g, BNP 443.29 pg/mL, TAT 14.2 ng/mL, D-dimer 2.91 mg/L; TNF-α 21.85 pg/mL. Thyroid function, OGTT and insulin release were normal; lipids remained high. A 24-h BP profile averaged 140/92 mmHg (range 128–154/82–108). She was diagnosed with severe EOPE with hypoalbuminemia, anemia, hyperlipidemia, and hypercoagulability. Management included a repeat magnesium sulfate protocol, intensified antihypertensives (labetalol 100 mg q8h + nifedipine 30 mg qd), diuresis/oncotic support (furosemide 20 mg IM + albumin 40 g IV), continued aspirin + fondaparinux, and supportive care. Ultrasound at 30^+4^ weeks showed Twin A ~1655 g and Twin B ~1416 g, with normal Dopplers and amniotic fluid.

Immune testing and immunomodulatory regimen (31 + 1 to 32 + 0 weeks). Given refractory progression under standard management (including optimized antihypertensives, magnesium sulfate, albumin/diuresis, and thromboprophylaxis), comprehensive immune testing was performed at 31 + 1 weeks (2024-12-20). Results were ANA weak positive, IgG 7.29 g/L (low), complement C4 76.30 mg/L (low), ESR 37 mm/h, and lymphocyte subset skewing (B cells 5.66%↓; total T cells 79.36%↑; absolute lymphocytes 1.173×10^9^/L↓). These findings were obtained after severe EOPE was established and are descriptive in this single case; they do not define a validated EOPE subtype. After multidisciplinary consultation (obstetrics, rheumatology/immunology, and nephrology) and informed consent for off-label biologic use in pregnancy, an immunomodulatory regimen was started: prednisone 10 mg qd, hydroxychloroquine 0.2 g bid, and certolizumab pegol 200 mg SC (single dose). Certolizumab was selected because it lacks an Fc region and has minimal placental transfer; a single dose was used as a conservative exposure-minimizing strategy in this off-label setting. No fever or infectious symptoms were documented at initiation, and close maternal–fetal monitoring continued. Over the ensuing 5–6 days, BP improved to 100–120/60–80 mmHg, edema regressed, and 24-h protein decreased to 4.99 g by 32 + 0 weeks (2024-12-26). She was discharged at 32 + 0 weeks on labetalol 100 mg q8h, aspirin 100 mg qd, fondaparinux 2.5 mg qd, prednisone 10 mg qd, and hydroxychloroquine 0.2 g bid, with close surveillance planned.

### Third admission and fetal surveillance (33^+0^ to 35^+0^ weeks)

At 33^+0^ weeks (2025-01-02), ultrasound showed growth discordance (Twin A ~34 weeks ≈2210 g; Twin B ~31+ weeks ≈1478 g) and cardiac changes in Twin A (increased cardiothoracic ratio, right-sided enlargement, wall thickening), raising concern for TTTS vs. selective fetal growth restriction (sFGR). Repeat high-level ultrasound the same day favored sFGR; both amniotic pockets were within normal ranges on expert scan. Maternal BP was 135/86 mmHg on labetalol; edema was minimal. Labs showed Hb 97 g/L, albumin 31.6 g/L, 24-h protein 4.52 g, and persistently elevated coagulation activation (TAT 11.0 ng/mL; D-dimer 2.53 mg/L; fibrinogen 3.43 g/L). She declined transfer to a provincial center and was admitted for intensive monitoring (daily NSTs and serial Dopplers).

By 33^+4^ weeks (2025-01-06), ultrasound documented elevated umbilical artery resistance in Twin B (S/D 3.9) and fetal tachycardia 171 bpm, supporting progressive placental insufficiency for Twin B. On 34^+3^ weeks (2025-01-12), maternal labs showed albumin 24.6 g/L and a rise in 24-h protein to 7.07 g; BP remained controlled at 124/71 mmHg, but edema increased (++) and fibrinogen was high (9.60 g/L). Albumin replacement and diuresis were repeated.

### Delivery (35^+0^ weeks; 2025-01-16)

**Figure 1:**
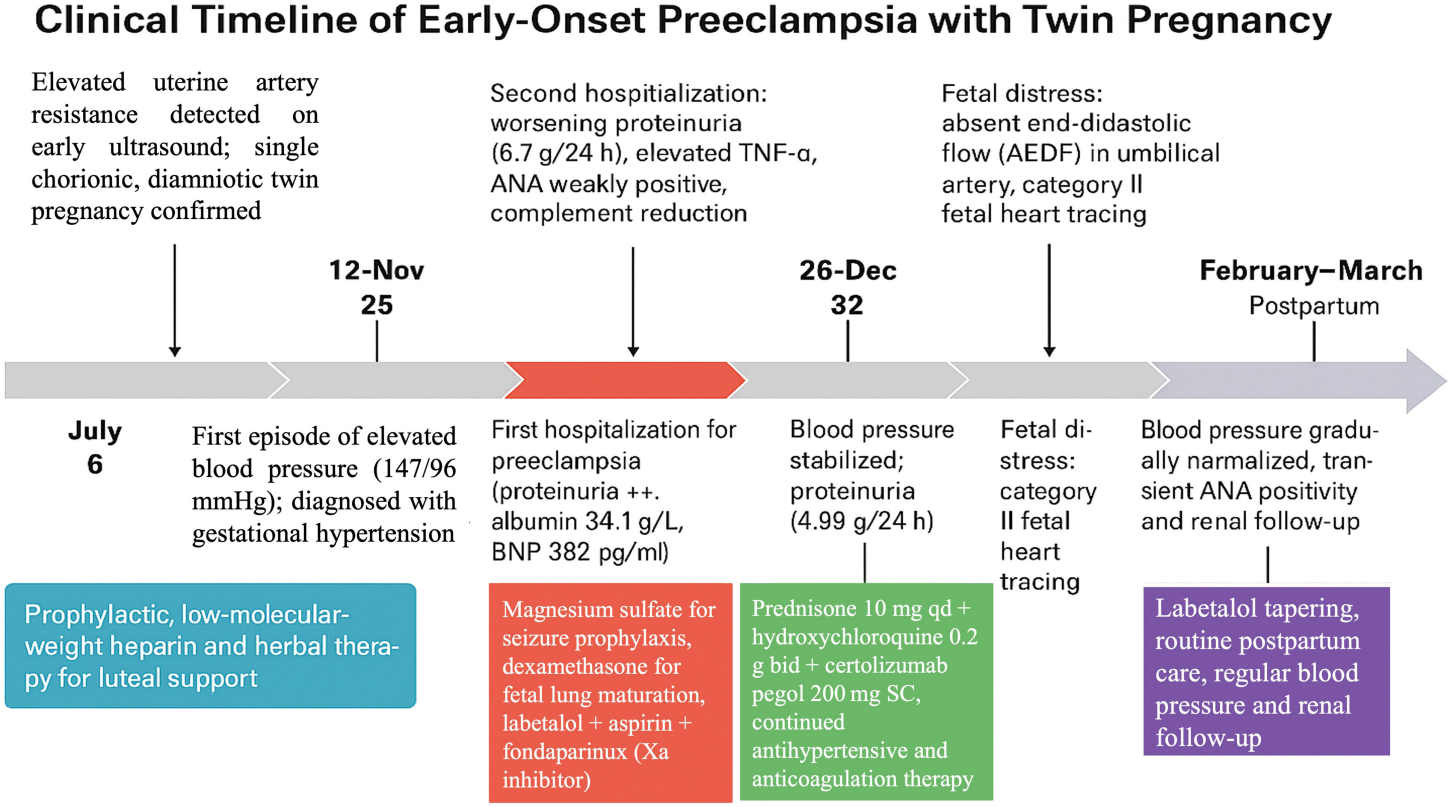
Clinical timeline of early−onset preeclampsia with twin pregnancy.

At 35^+0^ weeks, fetal testing deteriorated: absent end-diastolic flow (AEDF) in Twin B’s umbilical artery and non-reassuring fetal heart tracing (Category II) were documented. Maternal BP was 123/67 mmHg; 24-h protein rose to 10.74 g; albumin 29.2 g/L; liver enzymes and platelets remained within reference ranges. Given fetal compromise with sFGR Type II/AEDF and relapsing maternal proteinuria, an urgent cesarean was performed. Intraoperatively, amniotic fluid was clear for both twins. Twin A had a single nuchal cord; cord length and caliber were normal. Twin B had a hypercoiled, thin umbilical cord (~1.5 cm diameter) with torsion and marginal placental insertion. Multiple placental infarcts were visible. Neonatal outcomes: two live preterm infants were delivered and transferred to NICU (A-infant, first delivered, corresponded to prenatal Twin B, 1620 g, Apgar 9/9; B-infant, second delivered, corresponded to prenatal Twin A, 2340 g, Apgar 9/9). A chronological summary of key clinical events and interventions is presented in **Table 1** and **Figure 1**.

**Table 1:** Summary of key clinical events and interventions.

Date (2024–2025)	Gestational age (weeks)	Key clinical events	Main interventions
July	6	Elevated uterine artery resistance detected on early ultrasound; single chorionic, diamniotic twin pregnancy confirmed	Prophylactic low-molecular-weight heparin and herbal therapy for luteal support
12-Nov	25	First episode of elevated blood pressure (147/96 mmHg); diagnosed with gestational hypertension	Initiated labetalol 100 mg bid + aspirin 75 mg qd + vitamin D + iron supplement
28-Nov	28	Persistent hypertension despite treatment (SBP 140–150 mmHg; DBP 90–100 mmHg)	Increased labetalol dosage to tid; continued antiplatelet and vitamin therapy
4-Dec	29	First hospitalization for preeclampsia (proteinuria ++, albumin 34.1 g/L, BNP 382 pg/mL)	Magnesium sulfate for seizure prophylaxis, dexamethasone for fetal lung maturation, labetalol + aspirin + fondaparinux (Xa inhibitor), iron supplementation
December 11–20	30–31	Second hospitalization: worsening proteinuria (6.7 g/24 h), elevated TNF-α, ANA weakly positive, complement reduction	Prednisone 10 mg qd + hydroxychloroquine 0.2 g bid + certolizumab pegol 200 mg SC, single dose, albumin infusion, diuretics, continued antihypertensive and anticoagulation therapy
26-Dec	32	Blood pressure stabilized; proteinuria decreased (4.99 g/24 h)	Discharged on maintenance therapy (labetalol + aspirin + fondaparinux + immunotherapy)
2-Jan	33	Third hospitalization for suspected selective intrauterine growth restriction (sIUGR)/twin-to-twin transfusion syndrome	Continuous monitoring; maintained antihypertensive, anticoagulant, and immunomodulatory regimen
16-Jan	35	Fetal distress: absent end-diastolic flow (AEDF) in umbilical artery, category II fetal heart tracing	Emergency cesarean delivery; delivered two live preterm infants (A-infant 1620 g; B-infant 2340 g)
February–March	Postpartum	Blood pressure gradually normalized; transient ANA positivity and mild hyper-IgM	Labetalol tapering, routine postpartum care, regular blood pressure and renal follow-up

Table 1 summarizes the chronological clinical course, key diagnostic findings, and therapeutic interventions in a 22-year-old woman with early-onset preeclampsia and monochorionic diamniotic twin pregnancy.

### Placental pathology

The fused monochorionic–diamniotic placenta showed villous congestion, old infarctions with patchy calcifications, and fibrinoid deposition of the maternal basal plate—lesions consistent with maternal vascular malperfusion in preeclampsia. Umbilical cords were three-vessel (2A1V) bilaterally; prenatal Twin B’s cord insertion was marginal. These findings matched the clinical picture of EOPE with selective placental insufficiency affecting Twin B.

### Neonatal course

A-infant: Presented with weak cry and poor responsiveness; immediate blood glucose 0.6 mmol/L; blood gas pH 7.28, HCO_3_ 19.5 mmol/L, BE −5.9 mmol/L. Diagnoses included congenital pneumonia, type I respiratory failure, neonatal hypoglycemia, prematurity, low birth weight, and mixed acid–base disorder. Management: NIPPV, IV nutrition, and ceftazidime; phototherapy for hyperbilirubinemia on day 3. Cranial ultrasound noted periventricular echogenicity considered hypoxia related. (Culture results were not available in the records reviewed).

B-infant: Initial blood glucose 3.1 mmol/L; chest radiograph showed diffuse granular opacities and reduced lucency consistent with neonatal pneumonia/RDS; blood gas pH 7.35 with low PCO_2_ and mild hypoxemia. Management: noninvasive ventilation (then NIPPV), IV fluids, ceftazidime, and partial parenteral nutrition due to poor enteral feeding. Cranial ultrasound was unremarkable. Both infants recovered progressively and were discharged home after NICU care (A-infant at corrected 37 + 2 weeks, 2.08 kg; B-infant at corrected 36 + 4 weeks, 2.48 kg).

### Maternal postpartum course and follow-up

Post-cesarean care included prophylactic antibiotics (second-generation cephalosporin), uterotonics (carbetocin), seizure prophylaxis with magnesium sulfate, and LMWH 4000 U/day for days 1–5. By postoperative day 3, labs showed Hb 99 g/L, platelets 123×10^9^/L, albumin 27.6 g/L; urine protein had decreased to + on dipstick; pelvic/abdominal ultrasound was unremarkable. BP ranged 95–120/60–85 mmHg; the patient recovered well and was discharged. At day 10 postpartum, urine protein was 2+ and clinic BP 140–150/90–95 mmHg; labetalol 200 mg/day + nifedipine 30 mg/day were prescribed. At day 21, BP was ~145/90 mmHg with continued therapy. At day 49, BP improved to 130–140/80–90 mmHg; urinalysis was negative for protein and 24-h urine protein was 0.23 g; coagulation markers normalized (D-dimer 0.59 mg/L; fibrinogen 2.63 g/L; TAT 3.4 ng/mL). Immunology at that visit showed ANA positive (1:100, speckled); ENA panel negative; mild IgM elevation (3.77 g/L); total T-lymphocyte fraction mildly high; cardiac echo normal; pelvic ultrasound normal. Antihypertensives were tapered to labetalol 150–200 mg/day per home BP readings (generally 122–135/94–98 mmHg). At 3 and 6 months postpartum, telephone follow-up recorded normotension without medication and good maternal well-being twins were mixed-fed and developing appropriately. At 8 months, mother and infants remained well without new issues.

### Synopsis

This MCDA twin pregnancy progressed to severe EOPE with early hypertensive onset, nephrotic-range proteinuria, hypoalbuminemia, and hypercoagulability, with concurrent immune-activation markers (ANA positivity, low C4, elevated TNF-α, lymphocyte subset skewing) measured during the severe disease stage. An immune testing-informed immunomodulatory regimen (low-dose prednisone, hydroxychloroquine, and a single dose of a TNF-α inhibitor) was started after multidisciplinary discussion and was temporally associated with improved maternal BP/edema and a >50% decrease in proteinuria over one week; gestation then continued from 31^+1^ to 35^+0^ weeks, when delivery occurred for fetal indications (Type II sFGR with AEDF in Twin B). Placental infarctions and basal plate fibrinoid deposition were consistent with severe maternal vascular malperfusion. Both neonates and the mother had favorable short-term outcomes, and maternal proteinuria resolved by 6 weeks postpartum.

## Discussion

This case describes severe EOPE in a high-risk monochorionic diamniotic (MCDA) twin pregnancy, in which immune testing obtained during the severe disease stage showed immune-activation markers (elevated TNF-α, low complement C4, ANA positivity, and lymphocyte-subset skewing). After initiation of a pregnancy-compatible immunomodulatory regimen (prednisone, hydroxychloroquine, and a TNF-α inhibitor) alongside ongoing standard management, maternal clinical parameters were followed by improvement, and the pregnancy continued for approximately 3–4 additional weeks before delivery for fetal indications (sFGR type II with AEDF) (9). These observations are hypothesis-generating; causality and mechanism cannot be inferred from a single case with concurrent interventions.

### Immune dysregulation in EOPE: background and uncertainty

EOPE is increasingly framed as a syndrome initiated by impaired spiral-artery remodeling and placental ischemia, which provoke release of inflammatory mediators that injure the maternal endothelium. A robust literature documents heightened innate and adaptive immune activation in preeclampsia: elevated TNF-α, IL-6/IL-8, neutrophil activation/NETs, diminished Treg activity, and complement pathway dysregulation, all of which correlate with disease severity and earlier onset (10, 11). These mechanisms plausibly connect the placenta to maternal hypertension, proteinuria, and coagulopathy, and they provide therapeutic entry points beyond symptomatic control of blood pressure or seizure prophylaxis (9). In particular, complement abnormalities (e.g., reduced C4 and excess activation) recur across EOPE cohorts and overlap with thrombo-inflammatory obstetric syndromes, supporting the concept that selective immune blockade might stabilize a subset of patients long enough to gain fetal maturity (12). In our patient, biomarkers (high TNF-α, low C4, ANA+) were interpreted as immune-activation markers; however, because they were measured after severe EOPE was established, they may be secondary to disease severity and are descriptive in this single case (13).

### Rationale and temporal association of immune testing-informed immunomodulatory “bridging”

Given refractory EOPE at ~31 weeks (24-h protein 10.78 g, persistent edema) under standard care, we started low-dose prednisone (10 mg/day) for broad anti-inflammatory effect, hydroxychloroquine (HCQ 200 mg bid) for TLR/cytokine modulation and potential endothelial benefit, and a TNF-α inhibitor to address the markedly elevated TNF-α. In the subsequent days, blood pressure improved (100–120/60–80 mmHg), edema regressed, and proteinuria decreased by >50% (to 4.99 g/24 h within a week), coinciding with clinical stabilization that allowed outpatient management at 32^+0^ weeks. Gestation subsequently continued until 35^+0^ weeks, when delivery proceeded for fetal rather than maternal indications (Twin B AEDF with category II tracing). Because this is a single case with multidrug therapy and concurrent standard interventions, these changes should be interpreted as temporal association rather than attribution (14, 15).

### Why target TNF-α, and which agent is pregnancy-compatible?

TNF-α has a central role in EOPE pathobiology, impairing trophoblast invasion, amplifying oxidative stress, and driving endothelial dysfunction; higher maternal TNF-α associates with more severe disease (11, 16). Among TNF inhibitors, certolizumab pegol is distinctive because it lacks an Fc region and demonstrates negligible placental transfer in the CRIB pharmacokinetic study—supporting use when anti-TNF therapy is considered during pregnancy (17, 18). Beyond pharmacokinetics, emerging prospective data (IMPACT trial) in APS, a thrombo-inflammatory placental disorder, suggest add-on certolizumab markedly reduces placenta-mediated adverse outcomes compared with historical experience on heparin+aspirin alone, strengthening the broader concept of TNF blockade to protect the maternal–placental interface in high-risk pregnancies (19). Although our patient did not have APS, the overlap in effector mechanisms (excess TNF-α, endothelial injury, complement engagement) provides a biologic rationale to explore class-targeted TNF inhibition in selected EOPE cases with immune-activation markers—ideally within trials or with rigorous oversight (20, 21).

### Where does hydroxychloroquine fit?

HCQ is widely used in pregnancy for SLE and related autoimmune conditions, with accumulating evidence of maternal and fetal safety and signals of reduced hypertensive complications/preeclampsia in autoimmune cohorts (22–24). Recent multicenter studies show HCQ use in SLE pregnancies associates with lower rates of preeclampsia and preterm birth and fewer disease flares, consistent with its TLR- and cytokine-modulating effects that can temper endothelial inflammation (22, 25, 26). In our case, HCQ was part of a multimodal regimen (with prednisone, TNF inhibition, optimized antihypertensives, anticoagulation, and intermittent oncotic/diuretic support) that was temporally associated with improvement; its independent contribution cannot be determined (27).

### How does this compare with other immune-targeted or disease-modifying strategies?

Investigational approaches in severe preterm preeclampsia include complement inhibition (e.g., eculizumab) and apheresis to lower circulating sFlt-1. Case reports and small series suggest eculizumab can temporarily stabilize HELLP/PE with thrombotic microangiopathy features, sometimes extending pregnancy by ~2–3 weeks, while apheresis strategies typically extend gestation by days to a week in early-severe disease—beneficial but logistically intensive (14, 28, 29). Our observation of a multi-week interval between immunomodulatory initiation and delivery (without extracorporeal therapy) is therefore notable and hypothesis-generating, particularly in this severe EOPE case with immune-activation markers (30). Prospective, stratified studies are needed to compare TNF-targeting, HCQ, complement blockade, and anti-angiogenic removal strategies—alone or in combination—and to define biomarkers that enrich for responders.

### Twin-specific context: why the risk and why the severity?

Twin gestations, especially monochorionic twins, double or triple the risk of preeclampsia and often shift onset earlier, probably owing to larger placental mass, greater antigen load, and higher anti-angiogenic and inflammatory burden (5, 31, 32). In MCDA twins, distinguishing selective fetal growth restriction (sFGR) from twin-to-twin transfusion syndrome (TTTS) is crucial. Type II sFGR—defined by persistent absent/reversed end-diastolic flow (AEDF) in the umbilical artery—portends high fetal risk and is a common indication for delivery; these Doppler features were present in Twin B at 35 weeks, driving our decision for urgent cesarean independent of maternal status (33). The placenta in our case showed infarctions and basal plate fibrinoid deposition, classic for maternal vascular malperfusion in EOPE and consistent with the asymmetric placental supply that underlies sFGR (34).

### Safety, governance, and limitations

All immune-modifying measures were used with multidisciplinary oversight, and the off-label nature of TNF-α inhibition in pregnancy was discussed with the patient and family as part of informed consent. Consent for publication of de-identified clinical information was obtained. The selected agents have favorable pregnancy safety profiles in other indications (e.g., HCQ, certolizumab’s minimal transplacental passage), but controlled data in active preeclampsia remain limited; off-label use should be confined to expert centers with vigilant maternal–fetal monitoring (35). At the time of certolizumab initiation, no fever or infectious symptoms were documented, and both mother and infants were monitored clinically for infection during hospitalization and follow-up; however, this report cannot quantify infectious risk. Long-term infant immunologic outcomes (including infection susceptibility and vaccine responses) were not systematically assessed. This report has inherent limitations: it is a single case, causality cannot be inferred, and improvements may reflect combined effects of antihypertensives, anticoagulation, albumin/diuresis, and the natural waxing–waning of disease. We lacked serial immune measurements temporally aligned with clinical changes, angiogenic biomarkers (sFlt-1/PlGF), and functional immune assays, and we cannot disentangle the relative contributions of steroid vs HCQ vs anti-TNF. Finally, outcomes were not assessed using a pre-specified interventional endpoint. Overall, the observed sequence is hypothesis-generating and does not establish efficacy.

Beyond ethical oversight, safety considerations—particularly the risk of infection associated with biologic immunomodulation—are central when TNF-α inhibitors are considered during pregnancy. Safety considerations are central because TNF-α inhibition for EOPE is off-label. We therefore emphasize that infection surveillance should accompany any pregnancy exposure to TNF inhibitors. Recent evidence does not demonstrate a statistically significant association between TNFi exposure during pregnancy and serious infections in mothers or offspring, although a modest increase in risk cannot be excluded, underscoring the need for careful maternal–neonatal monitoring and prudent interpretation in a single-case report (36, 37).

### Clinical implications and a path forward

Current guidelines rightly emphasize that the definitive treatment for preeclampsia is delivery, with expectant management reserved for <34 weeks and only in settings capable of intensive surveillance. Our observation suggests that in a severe EOPE case with immune-activation markers, initiation of pregnancy-compatible immunomodulation coincided with clinical stabilization and time gained to later gestation; however, validated immune phenotyping and prospective trials are required before such approaches can be recommended. Practically, future studies could evaluate whether a parsimonious immune panel (e.g., ANA, complements C3/C4, antiphospholipid antibodies, and, where available, cytokines) together with angiogenic markers (sFlt-1/PlGF) can help stratify risk and identify candidates for protocolized investigation of pregnancy-compatible agents (e.g., HCQ, carefully selected TNF blockade) with standardized maternal and neonatal endpoints. Our case supports the feasibility of this research direction and aligns with advances in APS where biologic therapy has begun to improve placenta-mediated outcomes (19).

## Conclusion

In summary, this MCDA twin pregnancy complicated by severe EOPE and concurrent immune-activation markers was followed by maternal clinical stabilization after initiation of a pregnancy-compatible immunomodulatory regimen, and gestation continued until delivery was required for fetal indications. The findings support the concept that preeclampsia involves both vascular and immune pathways, but they remain hypothesis-generating; addressing immune pathways in EOPE should be studied in stratified, protocolized settings before any causal claims or clinical recommendations are made.

## Data Availability

The original contributions presented in the study are included in the article/supplementary material. Further inquiries can be directed to the corresponding authors.
